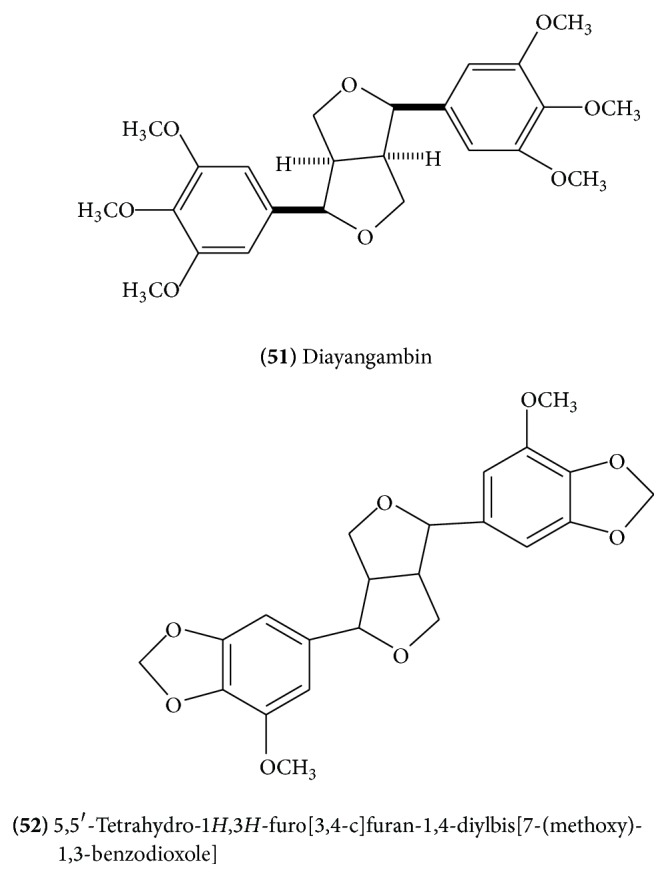# Pharmacology and Phytochemistry of Oleo-Gum Resin of *Commiphora wightii* (Guggulu)

**DOI:** 10.1155/2015/138039

**Published:** 2015-10-26

**Authors:** Prerna Sarup, Suman Bala, Sunil Kamboj

**Affiliations:** MM College of Pharmacy, Maharishi Markandeshwar University, Mullana, Ambala, Haryana 132 037, India

## Abstract

Guggulu is an oleo-gum resin which exudes out as a result of injury from the bark of *Commiphora wightii* (Arnott) Bhandari [syn.  *Commiphora mukul* (Hook. Ex Stocks) Engl; *Balsamodendron mukul* (Hook. Ex Stocks); Family, Burseraceae]. It has been used in the *Ayurveda* since time immemorial for the treatment of variety of disorders such as inflammation, gout, rheumatism, obesity, and disorders of lipids metabolism. It is a mixture of phytoconstituents like volatile oil which contains terpenoidal constituents such as monoterpenoids, sesquiterpenoids, diterpenoids, and triterpenoids; steroids; flavonoids; guggultetrols; lignans; sugars; and amino acids. This review is an effort to compile all the information available on all of its chemical constituents which are responsible for its therapeutic potential. The wild occurrence of this species is restricted mainly to the dry regions of Rajasthan and Gujarat States of India, and the bordering regions of Pakistan. Oleo-gum resin, guggulu, tapped from the stems of this species, is consumed in high volumes by the Indian herbal industries. There has been a decline in its wild population over the last several decades, as a result of habitat loss and degradation, coupled with unregulated harvesting and tapping of oleo-gum resin. This species is consequently assessed as Critically Endangered and enlisted in the IUCN red list of threatened species.

## 1. Introduction

Guggulu consists of oleo-gum resin obtained as an exudate from the tapping of stem and branches of* Commiphora wightii* (Arnott) Bhandari [syn.* Commiphora mukul *(Hook. Ex Stocks) Engl;* Balsamodendron mukul* (Hook. Ex Stocks); Family, Burseraceae]. The plant is commonly known as guggul tree and is found in arid areas of India, Bangladesh, and Pakistan. In India, it is found in Rajasthan, Gujarat, Assam, Madhya Pradesh, and Karnataka. It is a small, bushy tree with thorny branches and produces a yellowish gum resin (guggulu) in small ducts located throughout its bark. The trees are tapped by making an incision on the bark. The resin, which flows out, is allowed to harden before it is collected. The tree is tapped from November to January and the resin is collected through May to June. A guggul tree yields between 250 to 500 g of dry resin during each collection season [1, 2].

In Indian traditional system of medicine, guggulu has been used for thousands of years in the treatment of arthritis, inflammation, gout, rheumatism, obesity, and disorders of lipids metabolism [3]. It is known by different names like guggula, guggul, guggal, gugar, and Indian bdellium [4].

Guggulu occurs in vermicular pieces of pale yellow or brown coloured mass with aromatic odour and bitter astringent taste; when fresh it is viscid and golden coloured. It should produce not more than 5 percent of total ash and 1 percent of acid-insoluble ash. It yields not less than 27 percent of alcohol-soluble matter and not less than 53 percent of water-soluble matter. The genuine samples of guggulu contain 1 percent of volatile oil [2] and between 1.0 and 1.5 percent of guggulsterones (*Z* and *E*) [3].

*Guggulu Shodhana (shodhan vidhi).* It has been mentioned in Ayurvedic texts that administration of raw guggulu may lead to skin rashes, irregular menstruation, diarrhoea, headache, mild nausea, and, with very high doses, liver toxicity [5]. In order to overcome unwanted effects of raw guggulu, Ayurveda describes a number of purification processes (*shodhan vidhi*) using different fluids (dravyas), which not only take care of the adverse effects but also enhance the therapeutic activity. According to Ayurvedic texts, guggulu must be purified before incorporating into formulations [1]. During a process of shodhan, guggulu is treated with specific materials of biological origin, for example, herbal juices, cow urine, and cow milk. It is possible that some of the properties (chemical and biological) of shodhan materials are added to guggulu during the purification process. It is also possible that some of the toxic or harmful constituents of raw guggulu are neutralized, detoxified, or removed during this process.

There are a large number of commercial polyherbal anti-inflammatory formulations which are using guggulu as the chief ingredient [6]. However, no study has been done to investigate the process of Ayurvedic purification and its probable effect on therapeutic efficacy except for one report which states that gastric irritancy of guggulu is reduced with purification [7].

During the process of purification, foreign matter is removed from raw guggulu manually and is then broken into small pieces. The broken mass is wrapped in a piece of cloth (called potli) and hanged into an inert container (called dola yantra) containing one of the recommended media which are gomutra (cow urine), triphala kasaya (decoction of triphala), vasapatra kasaya (decoction of* Adhatoda vasica* leaves), vasapatra savrasa (aqueous extract of* Adhatoda vasica* leaves), dugdha (milk), and water. The guggulu is kept immersed, while fluid is boiled till all the soluble matter of guggulu is dissolved in the purifying vehicle. The insoluble part of guggulu is taken out and discarded. Further boiling is continued till guggulu solution forms a soft mass. It is then poured out over a smooth wooden board smeared with cow ghee or castor oil and dried in the sun. The dried mass is called purified guggulu (suddh guggulu) [1].

## 2. Phytoconstituents of Guggulu

Guggulu contains diterpenoids, triterpenoids, steroids, long-chain aliphatic tetrols, aliphatic esters, ferulates, lignans, carbohydrates, and a variety of inorganic ions besides minor amounts of sesamin and other unidentified constituents.

### 2.1. Volatile Oil and Its Terpenoidal Constituents

#### 2.1.1. Monoterpenoids

The gum resin of* C. wightii *yields about 0.4% of essential oil by steam distillation and its chief components are myrcene, dimyrcene, and polymyrcene [8]. Other components of the oil are eugenol, d-limonene,  *α*-pinene, (±) linalool, cineole, *α*-terpineol, d-*α*-phellandrene, methylheptanone, bornyl acetate, (±) geraniol, and some other unidentified compounds [9].

#### 2.1.2. Sesquiterpenoids

The gum resin of* C. wightii* has been reported to contain bicyclic sesquiterpene, cadinene [9].

#### 2.1.3. Diterpenoids

**Figure 1:**
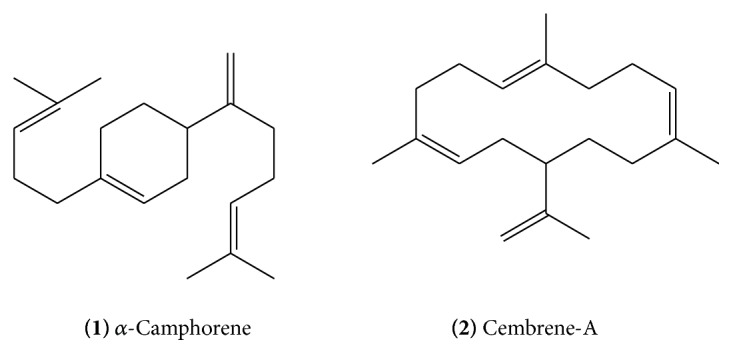


**Figure 2:**
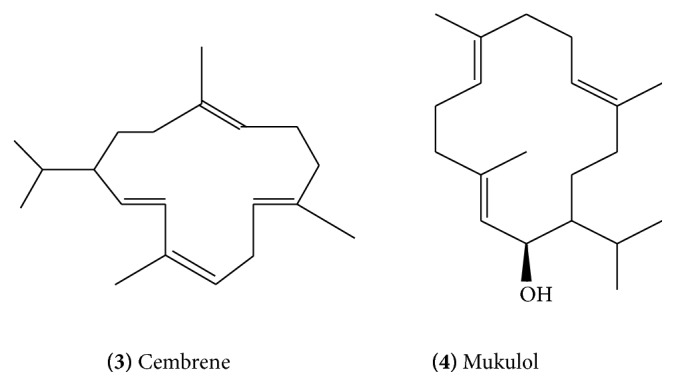


Diterpenoid constituents from guggulu include *α*-camphorene** (1) **(Figure 1) [10], cembrene-A** (2) **(Figure 1), cembrene** (3) **(Figure 2) [10], and other cembrenoids. Cembrene-A is one of the most elementary tetraenes derived from geranylgeranyl pyrophosphate by C-1 to C-14 cyclization.

**Figure 3:**
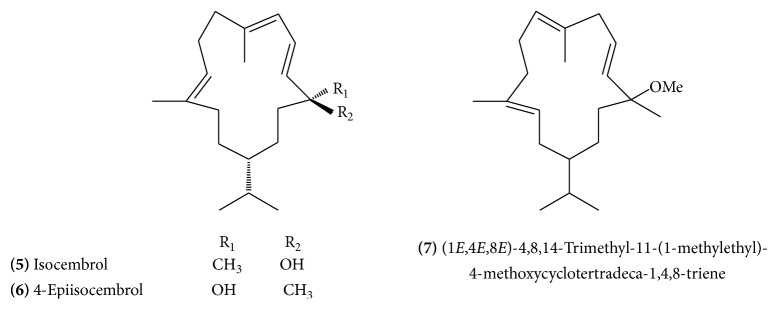


**Figure 4:**
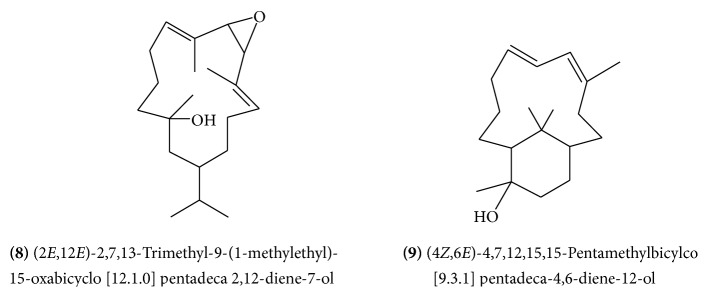


Mukulol (allylcembrol)** (4)** (Figure 2) is a new cembrane alcohol which was isolated from the aerial parts and also from the resin of guggulu [11, 12]. The allylcembrol structure was established by spectral analysis and mild dehydration which yielded cembrene. Other isolated cembrane type diterpenes include isocembrol** (5) **(Figure 3) and 4-epiisocembrol** (6) **(Figure 3). (1*E*, 4*E*, 8*E*)-4,8,14-Trimethyl-11-(1-methylethyl)4-methoxycyclotertradeca-1,4,8-triene** (7)** (Figure 3), (2*E*, 12*E*)-2,7,13-trimethyl-9-(1-methylethyl)-15-oxabicyclo [12.1.0] pentadeca-2,12-diene-7-ol** (8) **(Figure 4), and (4*Z*, 6*E*)-4,7,12,15,15-pentamethylbicylco [9.3.1] pentadeca-4,6-diene-12-ol** (9) **(Figure 4) were novel compounds obtained by bioassay-guided isolation from hexane-soluble portion of the methanol extract of guggulu [13].

#### 2.1.4. Triterpenoids

**Figure 5:**
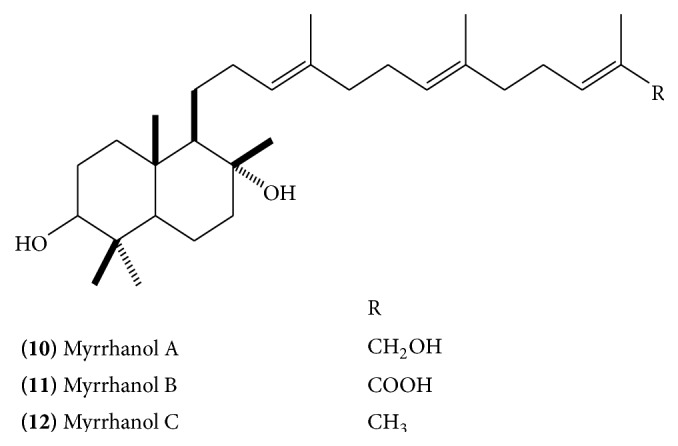


**Figure 6:**
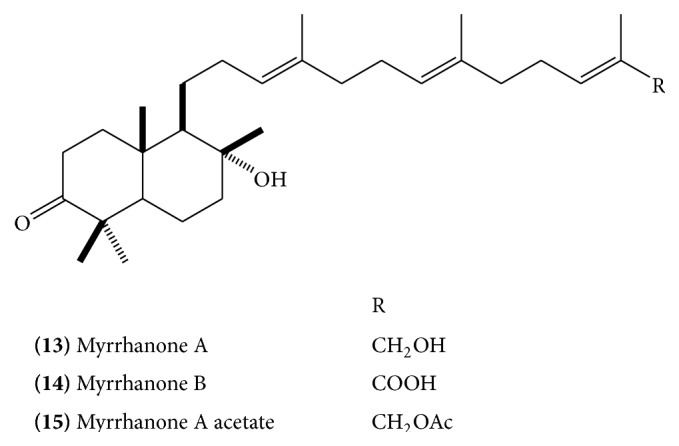


**Figure 7:**
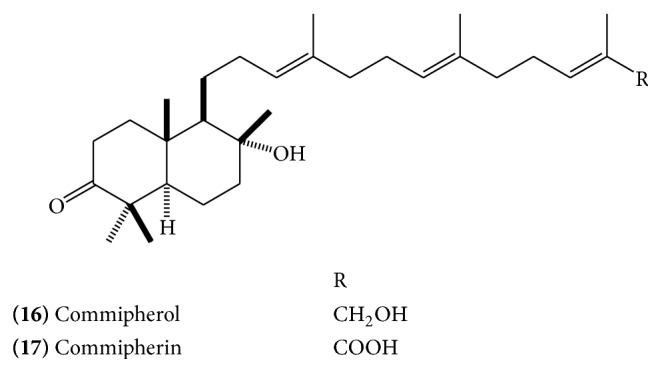


**Figure 8:**
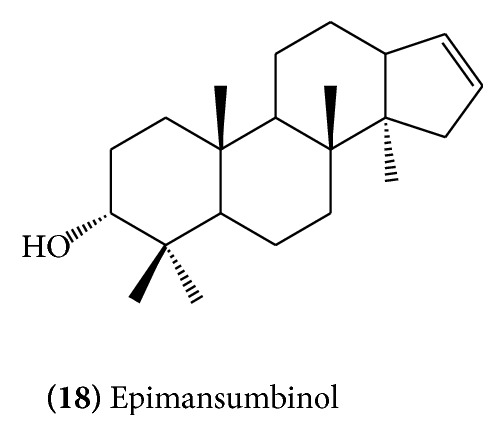


**Figure 9:**
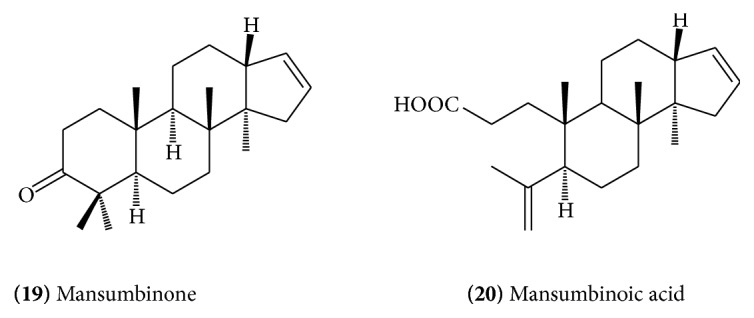


Polypodane-type triperpenes, myrrhanol A** (10)** (Figure 5), B** (11) (** Figure 5), and C** (12) (** Figure 5), myrrhanone A** (13)** (Figure 6), myrrhanone B** (14)** (Figure 6) [14, 15], myrrhanone A acetate** (15)** (Figure 6), commipherol** (16)** (Figure 7), commipherin** (17) (** Figure 7), and octanordammarane triperpenoid, epimansumbinol,** (18)** (Figure 8), have been isolated from the gum resin [16]. The isolation of two more triterpenoidal components has been reported, which are identified as mansumbinone** (19)** (Figure 9) and mansumbinoic acid** (20)** (Figure 9) [17].

**Figure 10:**
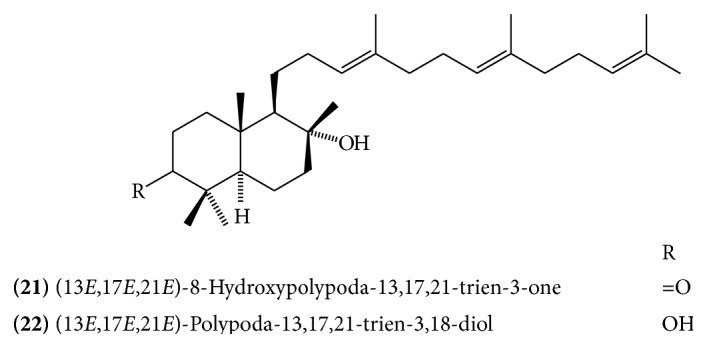


The absolute stereostructure of myrrhanol A was determined to be (3*S*, 5*S*, 8*R*, 9*R*, 10*S*)-3,8,30-trihydroxypolypoda-13*E*, 17*E*, 21*E*-triene. Myrrhanol B is 30-oic acid of myrrhanol A with altered stereostructure at C-5 (5*R* in contrast to 5*S* in myrrhanol A). Myrrhanone A and B are 3-keto analogue of myrrhanol A and B, respectively [15]. A myrrhanone derivative, (13*E*, 17*E*, 21*E*)-8-hydroxypolypoda-13,17,21-trien-3-one** (21) **(Figure 10), and a myrrhanol derivative, (13*E*, 17*E*, 21*E*)-polypoda-13,17,21-trien-3,18-diol** (22) **(Figure 10), have also been isolated [13].

### 2.2. Steroids

**Figure 11:**
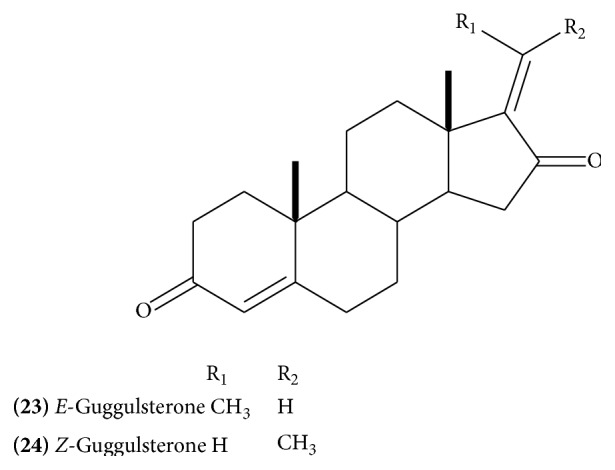


**Figure 12:**
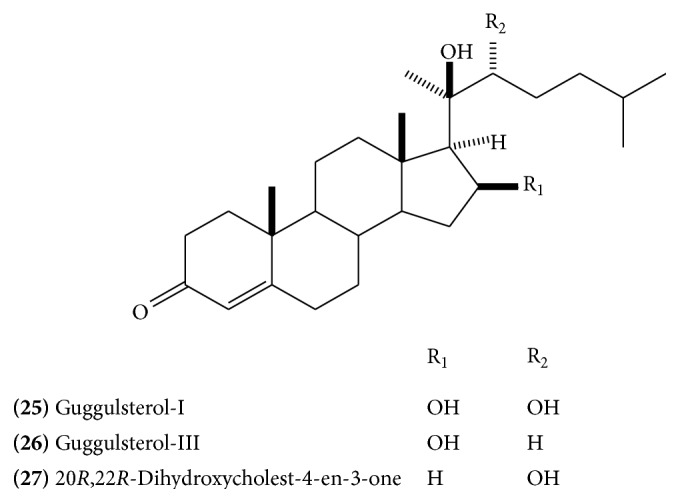


**Figure 13:**
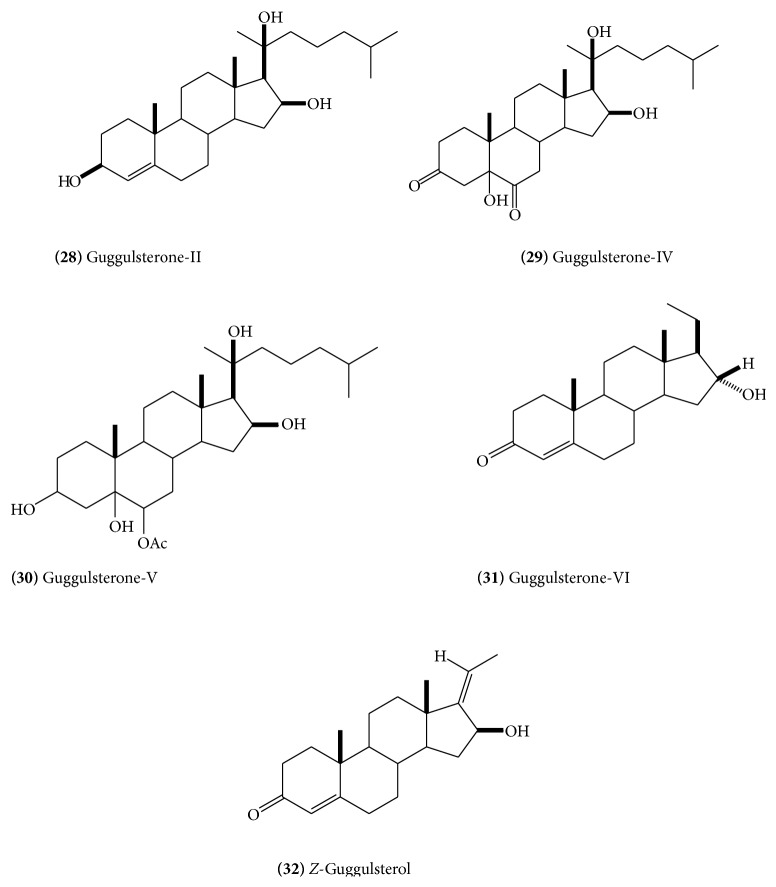


**Figure 14:**
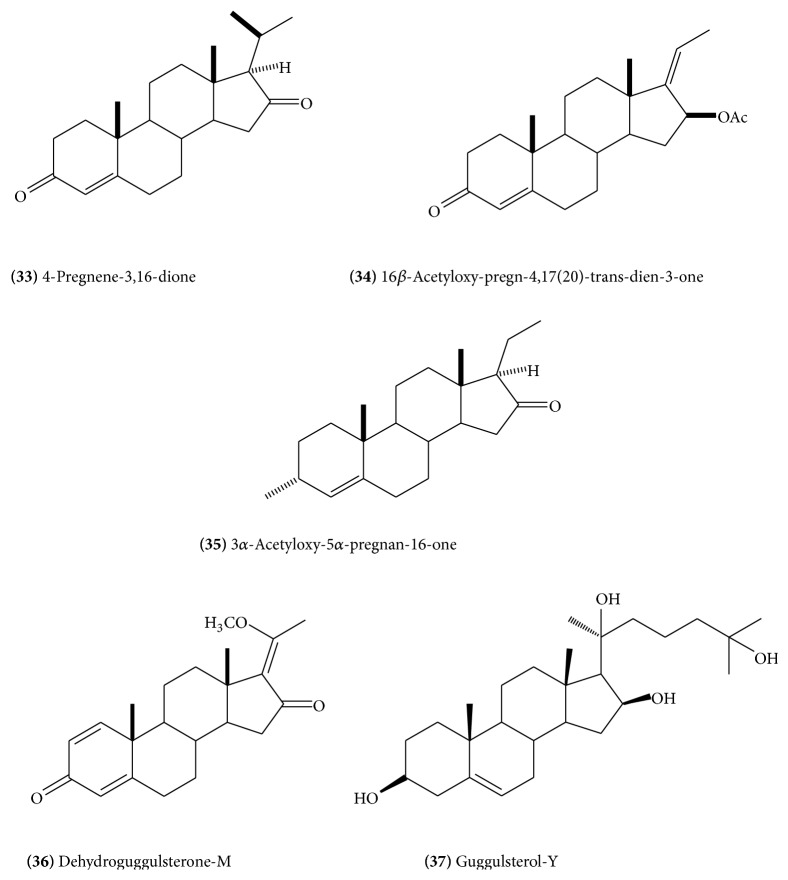


Isolation of several steroidal constituents has been reported from the gum resin. The major constituents include* E*-guggulsterone** (23) **(Figure 11),* Z*-guggulsterone** (24)** (Figure 11), guggulsterol-1** (25)** (Figure 12), guggulsterol-II** (26) **(Figure 12), guggulsterol-III** (27)** (Figure 12) [18], guggulsterol-IV** (28)** (Figure 13), guggulsterol-V** (29) **(Figure 13) [19], and guggulsterol-VI** (30)** (Figure 13) [20]. Other isolated steroids are 20*α*-hydroxy-4-pregnen-3-one** (31) **(Figure 13), 20*β*-hydroxy-4-pregnen-3-one, and 16*β*-hydroxy-4,17(20)-*Z*-pregnadien-3-one, which has been designated as* Z*-guggulsterol [20]. Progesterone and related steroids, 4-pregnene-3,16-dione** (32)** (Figure 13), (20*R*)-20-acetoxy-4-pregnene-3,16-dione,16*β-*acetyloxypregn-4,17(20)-*trans*-diene-3-one** (33) **(Figure 14), 3*α*-acetyloxy-5*α-*pregnan-16-one** (34) **(Figure 14), and 20*R*,22*R*-dihydroxycholest-4-en-3-one** (35) **(Figure 14), have also been isolated [16]. Cholesterol has also been reported. Three new and recently isolated steroids are guggulsterone-M, dihydro guggulsterone-M** (36) **(Figure 14), and guggulsterol-Y** (37) **(Figure 14) [17]. The steroidal constituents have been related with hypolipidemic and anti-inflammatory activities of the drug [13].

### 2.3. Flavonoids

**Figure 15:**
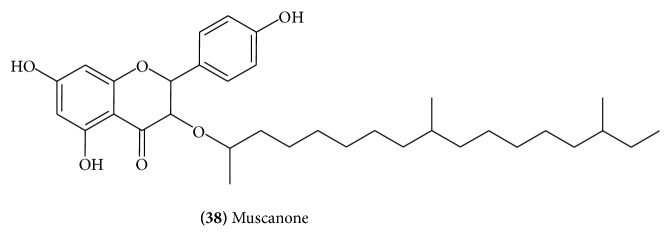


An ethanolic extract of trunk of* C. wightii* was separated on column packed with silica gel to give a new antifungal flavone named muscanone** (38) **(Figure 15) along with known naringenin. Muscanone was found to be active against* Candida albicans* in microbial sensitive assay [21].

**Figure 16:**
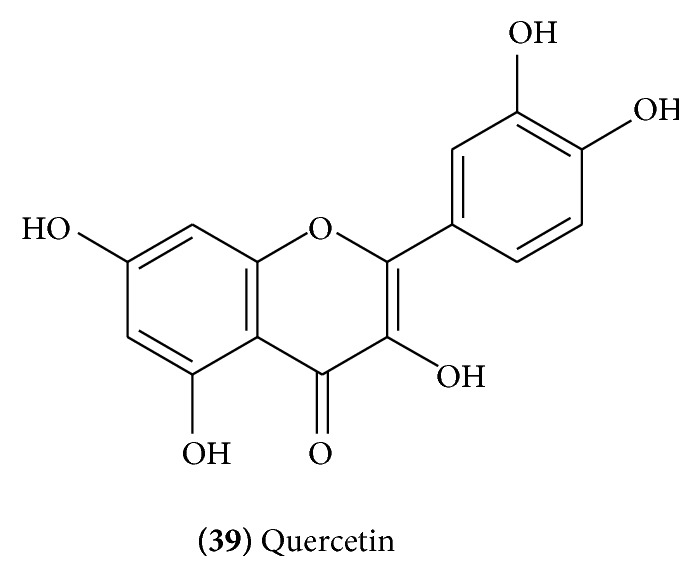


**Figure 17:**
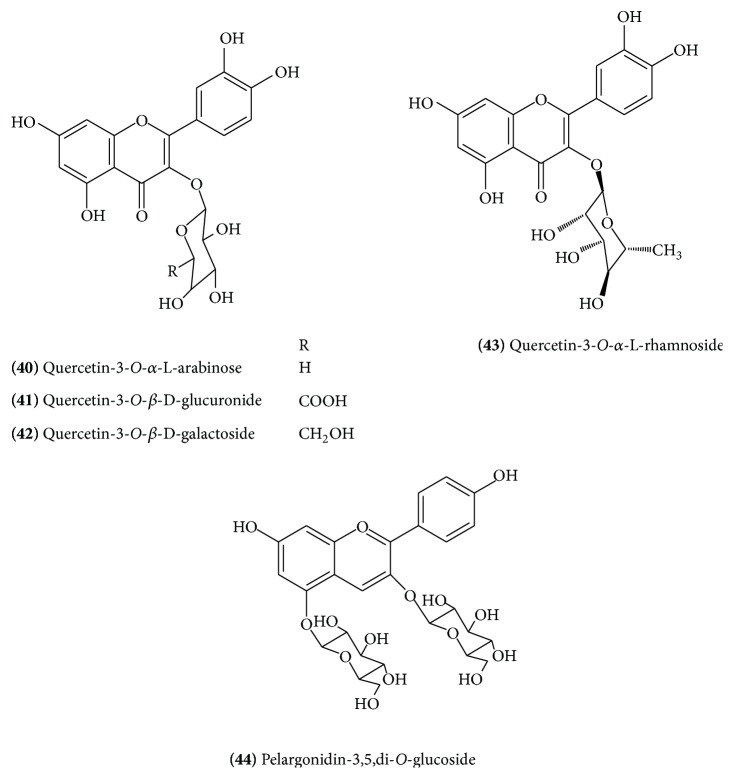


The major flavonoid components of the flowers of* C. mukul* were identified as quercetin** (39)** (Figure 16), quercetin-3-*O*-*α*-L-arabinose** (40)** (Figure 17), quercetin-3-*O*-*β*-D-glucuronide** (41)** (Figure 17), quercetin-3-*O*-*β*-D-galactoside** (42)** (Figure 17), quercetin-3-*O*-*α*-L-rhamnoside** (43)** (Figure 17), and pelargonidin-3,5,di-*O*-glucoside** (44)** (Figure 17) [22].

### 2.4. Guggultetrols

**Figure 18:**
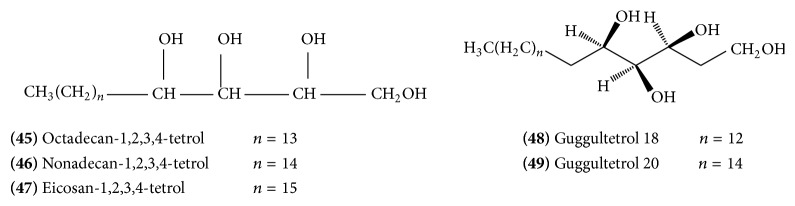


A crystalline material was isolated from the saponified gum resin which was characterized as a mixture of octadecan-1,2,3,4-tetrol** (45)** (Figure 18), nonadecan-1,2,3,4-tetrol** (46) (** Figure 18), and eicosan-1,2,3,4-tetrol** (47)** (Figure 18) with minor amount of other components, possibly lower (C-16 and C-17) and higher (C-21 and C-22) homologous tetrols. These compounds constitute a new class of naturally occurring lipids, guggultetrols. They are long-chain linear aliphatic tetrols with hydroxyl functions at C-1, C-2, C-3, and C-4 positions. Through derivatization and preparative GLC, guggultetrol-18** (48)** (Figure 18) and guggultetrol-20** (49)** (Figure 18) were obtained in pure form [23].

**Figure 19:**
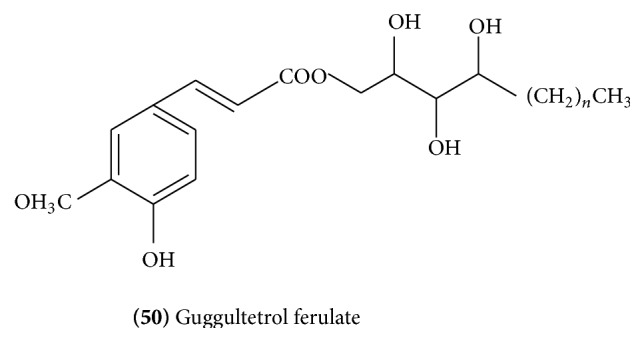


A mixture of two ferulates (*n* = 16, 17)** (50)** (Figure 19) with an unusual skeleton was found to be responsible for the cytotoxic action of the drug. They have been isolated from cytotoxic fraction of ethyl acetate extract of guggulu. It was identified as a mixture of esters based on homologous long-chain tetrols and acid [24].

### 2.5. Lignans

Two lignans, sesamin [18] and diayangambin** (51)** (Figure 20) [25], have been reported from guggulu. Also, 5,5′-tetrahydro-1*H*,3*H*-furo[3,4-c]furan-1,4-diylbis[7-(methoxy)-1,3-benzodioxole]** (52)** (Figure 20) has been reported from methanolic extract of guggulu [13].

### 2.6. Sugars

Complete hydrolysis of gum part of resin yielded L-arabinose, D-galactose, L-fructose (traces), and 4-*O*-methyl-D-glucuronic acid. Graded hydrolysis of the gum furnished an aldobiouronic acid [6-*O*-(4-*O*-methyl-*β-*D-glucopyranosyluronic acid)-D-galactose] [26]. Hydrolysis of methylated gum furnished 2,3,4,6-tetra-*O*-methyl-D-galactose, 2,3-di-*O*-methyl-L-arabinose, 2,3,4-tri-*O*-methyl-D-galactose, 2,4-di-*O*-methyl-D-galactose, and 2,3,4-tri-*O*-methyl-D-glucuronic acid in the ratio of 1 : 1 : 1 : 2 : 1. The provisional structure showed the gum to be a highly branched polysaccharide containing 1–6, 1–3, and 1–5 type of linkage [27].

### 2.7. Amino Acids

*C. mukul* was extracted with alcohol and the extract after removal of the solvent was partitioned between water and ether. The aqueous fraction was chromatographed and it showed the presence of various amino acids. The amino acids detected were cystine, histidine, lysine, arginine, aspartic acid, serine, glutamic acid, threonine, alanine, proline, tyrosine, tryptophan, valine, leucine, and isoleucine [28].

## 3. Traditional Uses of Guggulu

Guggulu has a long history of use in Ayurveda. The* Atharvaveda *is the earliest reference containing its medicinal and therapeutic properties [29]. Detailed description regarding its actions, uses, and indications and the varieties of guggul have been described in numerous Ayurvedic treatises including* Charaka Samhita* (1000 B.C.),* Sushruta Samhita* (600 B.C.) and* Vagbhata *(7th century A.D.). In addition, various medical lesions were written between the 12th and 14th centuries A.D. [30]. Guggulu has been used to treat obesity, osteoarthritis, rheumatoid arthritis, gout, facial paralysis, sciatica, constipation, haemorrhoids, liver disorders, inflammation, cyst, cervical lymphadenitis, coronary thrombosis, anaemia, diabetes, urinary calculus, increased frequency and turbidity of urine, and skin diseases [31, 32].

It has a wide range of usefulness in indigenous medicine. It is astringent and antiseptic and acts as a bitter, stomachic, and carminative when taken internally. Like all oleo resins, it causes increase in number of leucocytes and stimulates phagocytosis. It acts as a diaphoretic, expectorant, diuretic, uterine stimulant, and emmenagogue. The resin is used in the form of lotion for indolent ulcers and as a gargle in caries, spongy gums, pyorrhea, chronic tonsillitis, and ulcerated throat. Inhalation of the fumes from burnt guggulu is recommended in hay fever, acute and chronic nasal catarrh, chronic laryngitis, chronic bronchitis, and phthisis. It is an ingredient of ointment for ulcers [2].

## 4. Pharmacological Activity of Guggulu

### 4.1. Hypolipidemic Activity

The lipid lowering effect of guggulu with special reference to atherosclerosis and obesity (*medoraga*) was first reported in a doctorate thesis submitted to the Banaras Hindu University (BHU) in January 1966. Earlier to this work, guggulu was well known as an Ayurvedic drug for the treatment of various types of arthritis. This work was inspired by a rather obscure* shloka *in Sanskrit in the well-known Ayurvedic treatise* Sushruta Samhita*. The* shloka* deals in an extraordinarily lucid and scientific manner, with the etiology, pathogenesis, and treatment of obesity and associated lipid disorders and their complications. The hypolipidemic activity was shown in animals as well as in patients of obesity and hypercholesterolemia [33].

In carefully planned studies carried out (over a period of two years) on rabbits, in which hyperlipidemia was induced by feeding cholesterol (in hydrogenated vegetable oil), it was demonstrated for the very first time that crude guggulu could not only lower significantly the serum cholesterol in hypercholesterolemic rabbits but also protected these animals against cholesterol-induced atherosclerosis at the fatty streak stage. It also reduced the body weight of the animals. A similar trend to reduce significantly the serum cholesterol levels in patients with obesity and hypercholesterolemia was found in clinical studies with crude guggulu. The Central Drug Research Institute (CDRI), Lucknow, has been engaged in chemical, pharmacological, and clinical studies on guggulu [33].

Gugulipid, an ethyl acetate extract of the oleoresin, standardized at CDRI, has been marketed in India since 1988 as a hypolipidemic agent. It contains *Z*-guggulsterones and *E*-guggulsterones which are purported to be the compounds responsible for the hypolipidemic activity of the guggulu [34, 35]. Gugulipid contains not less than 4 percent and not more than 6 percent of guggulsterones (*Z*and *E*). The decision to use the ethyl acetate extract rather than two guggulsterones was primarily for commercial reasons and was also because of the fact that other components of the ethyl acetate extract showed synergistic (hypolipidemic) effect [33]. A number of clinical studies were carried out to confirm hypolipidemic activity of guggulu and gugulipid [36, 37]. The findings of multicentric clinical trials carried out with gugulipid at seven different centres in India coordinated in collaboration with CDRI confirmed the role of gugulipid as a hypolipidemic agent [36].

The hypolipidemic activity of guggulu and its various fractions has been studied in several animal models and clinical studies [1]. Initial studies reported that crude guggulu has encouraging hypolipidemic activity in rabbits [38]. This activity was also confirmed in other animals including white leghorn chicks, domestic pig, Presbytis monkey, and albino rat [1].

Studies were also conducted on the petroleum ether fraction (A), alkali washed neutral portion (B), and petrol-insoluble fraction (C) in healthy 8-week-old male white leghorn chicks in which hypercholesterolemia was induced by atherogenic diet fed for 2-3 weeks. All the fractions were found to lower the serum cholesterol, with fraction A being the most potent and fraction B being the least potent. None of the three fractions, however, was found to prevent the induction hypercholesterolemia in the chicks [38].

Further studies on the alcoholic extract and two pure compounds (a terpenoid and a steroid) isolated from the petroleum ether extract showed that the steroid was highly potent in lowering the serum cholesterol by 69.3 percent. The alcoholic extract could lower the cholesterol by 59.2 percent, whereas the terpenoid lowered it by 54.3 percent [38].

In another study, highly significant reduction in levels of mean serum cholesterol and triglyceride was observed in groups of animals receiving high-fat diet for one month along with guggulu, which clearly demonstrated its hypolipidemic activity. Additionally, administration of guggulu partially reversed the atherosclerosis in the aorta that was induced by high-fat diet [39].

Clinical studies on* C. mukul* showed its hypolipidemic effect and the outcome of change in lipid profile upon its administration. This study showed significant decrease in total cholesterol and LDL cholesterol after treatment with guggulu [40].

The hypolipidemic activity of the isomers *E*-guggulsterone and *Z*-guggulsterone has also been studied in animal models. Administration of guggulsterone (*Z* and *E*) significantly lowered serum lipid levels of rats with either triton (WR-1339) or cholesterol-induced hyperlipidemia [41].

Several mechanisms of action have been proposed for the hypolipidemic action of guggulu. Guggulu may decrease hepatic steroid production which ultimately increases the catabolism of plasma LDL cholesterol. Alternatively, the proposed active components of guggulu, guggulsterones *E* and *Z*, may increase hepatic binding sites for LDL cholesterol, thus increasing LDL clearance. However, recent findings have suggested that guggulsterone *E* and *Z* are highly efficacious antagonist of the farnesoid X receptor (FXR), a nuclear hormone receptor that is activated by bile acids, thus allowing increased cholesterol catabolism and excretion from the body [42, 43].

Guggulsterone and cembrenoids from* C. mukul* stem bark resin were shown to be specific modulators of two independent sites that are also modulated by bile salts to control cholesterol absorption and catabolism. Guggulsterone antagonized the chenodeoxycholic acid-activated nuclear farnesoid X receptor (FXR), which regulates cholesterol metabolism in the liver. The cembrenoids did not show a noticeable effect on FXR but lowered the cholate-activated rate of human pancreatic IB phospholipase A_2_ (hPLA2), which controls gastrointestinal absorption of fat and cholesterol [44].

### 4.2. Effect on Platelet Aggregation and Fibrinolytic Activity

The purified steroid mixture from guggulu completely inhibited ADP, adrenaline, or serotonin induced platelet aggregation. No difference was observed between the effectiveness of the steroid mixture and the purified guggulsterone *E* or *Z*. The effect of guggulsterones *E* and *Z* was very similar to the inhibitory effect of clofibrate. This finding has therapeutic value in myocardial infarction and thromboembolism [45].

The effect of guggulu on fibrinolysis and platelet adhesiveness in coronary heart disease was studied. Guggulu fraction A (pet ether extract) in daily dose of 1 g was administered to healthy individuals (group I) and to patients of coronary artery disease (CAD) (group II) for a period of 30 days. Serum fibrinolytic activity increased, while the platelet adhesive index decreased, which was statistically significant in healthy individuals and in CAD patients. In view of this, guggulu fraction A may be a useful therapeutic agent in the management of coronary artery disease [46].

### 4.3. Thyroid Stimulatory Activity

Administration of ethanolic extract of guggulu to the female albino mice for 15 days enhanced the triiodothyonine (T3) concentration and T3/T4 ratio, while no marked change in the concentrations of serum thyroxine (T4) was observed [47]. *Z*-Guggulsterone was shown to be responsible for the thyroid stimulatory action of guggulu. Administration of isolated *Z*-guggulsterone to rats led to significant increase in all thyroid function parameters, namely, uptake of iodine by the thyroid, enzymes involved in the synthesis of thyroid hormones, and tissue oxygen uptake, thus suggesting thyroid stimulatory action [48].

### 4.4. Anti-Inflammatory and Antiarthritic Activity

The results of several studies confirm anti-inflammatory and antiarthritic activities of guggulu [13, 15, 25, 49–52]. The 50 percent aqueous methanolic extract was found to exhibit an anti-inflammatory effect on adjuvant-induced air pouch granuloma in mice. The methanolic extract inhibited nitric oxide production in lipopolysaccharide activated mouse peritoneal macrophages [15]. A crystalline steroid was isolated from the petroleum ether extract and tested in rats for inhibition of inflammation induced by Freund's adjuvant. It inhibited the full development of the primary lesions in adjuvant arthritis and also reduced the severity of secondary lesions as compared with the untreated control group [53].

Guggulosomes prepared using guggul with ibuprofen by bath sonication and trituration methods were studied for anti-inflammatory activity. It was clearly shown that guggulosomes had more efficacy than ibuprofen and both guggul and ibuprofen had synergistic effect. The study proved that guggul could serve as a carrier for entrapping drugs and for their sustained release action [54].

Several animal studies have demonstrated the effectiveness of guggulu extract in standard osteoarthritis (OA) models. The authors had conducted both animal and clinical investigations of guggulu for OA prior to this study. The goal of this study was to determine the effectiveness of guggulu for reduction of pain, stiffness, and other symptoms that arise from OA [55].

### 4.5. Antioxidant Activity

The antioxidant property of guggulu helped stop the oxidation of cholesterol and subsequent hardening of the arteries, reduced the stickiness of platelet, and also lowered the risk of coronary artery disease [45]. It also enhanced the production of thyroxin and triiodothyronine; these hormones increase the metabolism of carbohydrates and protein synthesis and help in lowering the lipid activity [47].

The antioxidant activity was attributed to the presence of guggulsterones. It was tested* in vitro *against the formation of oxygen free radicals. The oxidation of human LDL induced by Fe^2+^ or by rat peritoneal macrophages caused marked formation of lipid peroxidation products. Guggulsterone (50 *μ*M) prevented the generation of thiobarbituric acid reactive substances and lipid hydroperoxide of low density lipoprotein in above system. However, it did not protect lipids against the formation of conjugated dienes, the initial step of lipid peroxidation cascade. Guggulsterone significantly inhibited the reaction of lipid peroxidation in liver microsomes challenged with Fe^2+^ and sodium ascorbate. Thus, the protective action of guggulsterone might also be due to free radical scavenging property. The metal chelating capacity of guggulsterone might be contributing to its antioxidant activity [56]. Also, the alcoholic extract of* C. mukul* exhibited antioxidant property [57].

### 4.6. Antiatherosclerotic Activity

LDL has been found to accumulate in atherosclerotic lesions and is the major source of the cholesterol accumulation in human foam cells. There is evidence that LDL oxidation is essential for atherogenesis and the antioxidants that prevent this oxidation may either slow down or prevent atherogenesis. Guggulsterones, the lipid-lowering components of guggulu, effectively inhibited* in vitro *LDL oxidation (as discussed under antioxidant action). Thus the combination of antioxidant and lipid-lowering properties of guggulu makes it especially beneficial against atherogenesis [58].

### 4.7. Cardioprotective Activity

Guggulsterones are shown to be effective cardioprotectives. Myocardial necrosis induced by isoproterenol in rats caused marked increase in serum creatine phosphokinase and glutamate pyruvate transaminase. Phospholipase, xanthine oxidase, and lipid peroxides were simultaneously enhanced in ischemic heart following depletion of glycogen, phospholipids, and cholesterol. Treatment with guggulsterone at a dose of 50 mg/kg significantly protected cardiac damage as assessed by the reversal of blood and heart biochemical parameters in ischemic rats [59].

### 4.8. Cytotoxic Activity

Ferulates, important bioactive constituents identified from the guggulu gum, were reported to play a significant role in* in vitro* cytotoxicity by decreasing the cell viability in MCF-7 (breast) tumor cells, PC-3 (prostate) tumor cells, and parental and transfected P 388 cells [49]. Therefore, ferulate compounds are used in the method for prevention and treatment of abnormal cell growth and proliferation of inflammation, neoplasia, and cardiovascular disease. Ethyl acetate extract showed significant* in vitro *cytotoxicity. A fraction showing cytotoxic activity was characterized as a mixture of two ferulates with an unusual skeleton by spectral and chemical methods. This fraction also showed moderate scavenging effect against 2,2-diphenyl-1-picryl hydrazyl (DPPH) radicals [60].

Treatment with gugulipid significantly inhibited the viability of human prostate cancer cell line LNCaP (androgen-dependent) and its androgen-independent variant (C-81) with IC_50_ of 1 *μ*M (24 h treatment), thus indicating its possible role in apoptosis and cancer prevention [60]. The results of this study indicated that guggulsterone inhibited proliferation of PC-3 cells in culture by causing apoptosis, whereas a normal prostate epithelial cell line is resistant to growth inhibition and apoptosis induction by this phytoconstituent. These observations provided rationale for further preclinical and clinical evaluation of guggulsterone for its efficacy against prostate cancer [61].

### 4.9. Antifertility Activity

Guggulu administered orally (2 and 20 mg/100 g body weight) to female rats decreased the weight of the uterus, ovaries, and cervix, whereas glycogen and sialic acid levels in these organs increased. This suggested that guggulu may be useful as an antifertility agent [62].

### 4.10. Skin Diseases

Administration of gugulipid was reported to be effective in the treatment of nodulocystic acne. A study in 21 patients found that gugulipid was as effective as tetracycline in the treatment. The patients with oily faces responded better to the gugulipid treatment [63].

### 4.11. Antihyperglycemic Activity

Administration of alcoholic extract of* C. mukul* at a dose of 200 mg/kg for 60 continuous days reduced plasma glucose levels in streptozotocin-induced diabetic rats [57]. A study showing effect of guggulsterone isolated from* C. mukul* in high-fat diet induced diabetic rats has also been reported. Different biochemical parameters like GTT, glycogen content, glucose homeostatic enzymes (like glucose-6-phosphatase and hexokinase), insulin release* in vivo*, and expression profiles of various genes involved in carbohydrate and lipid metabolism clearly demonstrated the hypoglycemic effect. The results suggested that guggulsterone has both hypoglycemic and hypolipidemic effects which can help cure type II diabetes [64].

### 4.12. Antimicrobial Activity

The volatile oil of* C mukul *was found to be highly effective against* Rhyzopertha dominica *which suggested its role as a fumigant. The ethanolic extract of* C. mukul* exhibited best antibacterial activity at 5 mg/mL against multidrug-resistant* Klebsiella pneumonia* [65]. An active compound, 5(1-methyl,1-aminoethyl)-5-methyl-2-octanone, of the methanolic extract of guggulu gum possessed significant antibacterial activity against Gram-positive bacteria and moderate activity against Gram-negative bacteria [66–68].

## 5. Safety and Toxicity

It has been mentioned in Ayurvedic texts that administration of raw guggulu may sometimes lead to skin rashes, irregular menstruation, diarrhoea, headache, mild nausea, and, with very high doses, liver toxicity [5]. In order to overcome the side effects of raw guggulu, Ayurveda describes a number of purification processes (shodhan vidhi) in different “dravyas,” that is, fluids, which not only take care of the adverse effects but also enhance the therapeutic activity [2]. It is also mentioned in Ayurvedic texts that guggulu must be purified before incorporation into herbal formulations. There are a large number of commercial polyherbal anti-inflammatory formulations which are using guggulu as the chief ingredient [52].

The clinical trials done with standardized gum guggul extracts reported transient side effects such as skin rashes, diarrhoea, and irregular menstruations [33]. A report also states that, out of 22 individuals receiving 2160 mg guggulu daily for 12 weeks, 10 persons experienced one or another side effect including gastrointestinal distress, fatigue, and skin rash [69]. Skin rashes have also been reported in other trials using 1-2 g guggulipid (ethyl acetate fraction) daily for a month. This study did not report any intestinal distress [70].

Although generally accepted as relatively safe, caution may be warranted during guggul consumption. There is little or no information on toxicity with the use of guggulu.

## 6. Conclusions

From this review article it is concluded that the resin of* Commiphora wightii*, guggulu, has emerged as a good source of the traditional medicines for the treatment of inflammation, arthritis, obesity, microbial infection, wound, pain, fractures, tumor, and gastrointestinal diseases. It is one of the oldest and the most prominent herbs in Ayurvedic medicine. Guggulu is a versatile drug and, because of its paranormal properties, it is very valuable in treating variety of disorders. Pharmacological results have validated the use of this plant in the traditional medicines. This plant contains a number of bioactive constituents including terpenoids, steroids, flavonoids, guggultetrols, lignans, sugars, and amino acids. Guggulsterones *E* and *Z* are the chief bioactive constituents of this resin and are endowed with immense pharmacological value. These conclusions could open a new window on the use of this plant in Ayurveda. This review clearly authenticates the Sanskrit definition of the term “guggul” which means one that protects against diseases. It is superbly reflected and proved by the diverse medicinal uses of this Ayurvedic drug.

*In vitro* studies and clinical trials help improve and advance medical care. They also assist health care professionals to direct resources to the strategies and treatments that would work best for a particular type of ailment. Although the use of guggulu in therapeutic doses appears to be safe and nontoxic more and more of such studies should still be conducted so that chances of any toxicity, if any, can be ruled out. It has also been mentioned that during the course of using guggulu one should avoid foods that are sour or bitter in taste, alcohol, excessive exercise, physical and mental strain, anger, and exposure to direct sunlight. Such data can only be validated when we would choose* in vitro* studies over* in vivo* studies.

Also, this plant is listed in IUCN list and thus rationale usage of the plant is the need of the hour so that we do not end up depleting this wonder drug of high therapeutic importance. Keeping this in view, stem, bark, and leaf of this plant should receive more attention so that the complete depletion on account of plant death due to tapping can be checked. This plant still possesses an unexplored potential and expansion of research materials would provide more opportunities for the discovery of novel bioactive principles from this plant.

## Figures and Tables

**Figure 20:**